# A simple and fast method for fixation of cultured cell lines that preserves cellular structures containing gamma-tubulin

**DOI:** 10.1016/j.mex.2018.02.003

**Published:** 2018-03-03

**Authors:** Maria Alvarado-Kristensson

**Affiliations:** Molecular Pathology, Department of Translational Medicine, Lund University, Jan Waldenströms gata 59, SE-205 02 Malmö, Sweden

**Keywords:** Fixation technique, Immunofluorescence, Cell lines, Cytoskeleton

## Abstract

When using fluorescence microscope techniques to study cells, it is essential that the cell structure and contents are preserved after preparation of the samples, and that the preparation method employed does not create artefacts that can be perceived as cellular structure/components. γ-Tubulin forms filaments that in some cases are immunostained with an anti-γ-tubulin antibody, but this immunostaining is not reproducible [1]. In addition, the C terminal region of γ-tubulin (green fluorescence protein tagged [GFP]-γ-tubulin^334––449^) forms cytosolic GFP-labeled structures, which can easily be imaged in live cells but are not preserved in fixed cells [1,2]. The purpose of this study was to identify a fixation technique that preserves cellular constituents containing γ-tubulin.

• This protocol describes a method that preserves γ-tubulin-containing structures in fixed cells.
• The technique entails two-step fixation. A pre-fixation step using paraformaldehyde is followed by a final fixation and permeabilization step performed at −80 °C.
• In comparison with other methodology for fixation [[3], [4], [5]], the technique presented here uses a short pre-fixation step with a mixture of paraformaldehyde and sucrose followed by a short fixation/permeabilization step with a mixture of methanol and acetone at −80 °C.

## Graphical abstract

**Figure:**
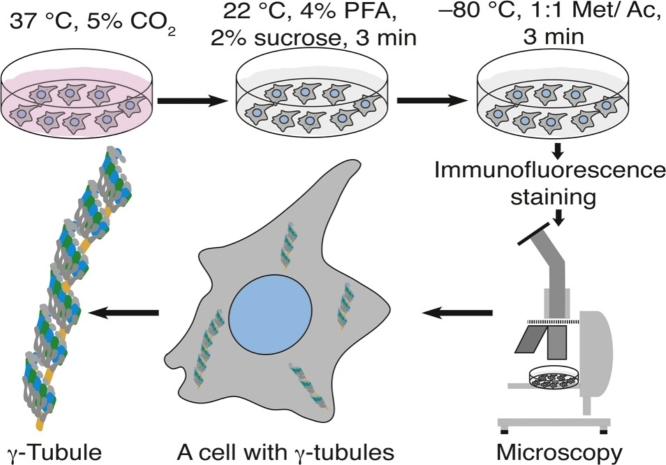

## Specifications table

**Table:** 

Subject area	*Select one of the following subject areas:*
	• *Molecular Biology*
More specific subject area	*Microscopy*
Method name	Fixation technique
Name and reference of original method	https://doi.org/10.1016/j.bbamcr.2017.10.008
Resource availability	*http://www.sciencedirect.com/science/article/pii/S0167488917302835?via%3Dihub*

## Method details

Live imaging followed by fixation of the imaged cells

*Step 1: preparation of stable cell lines*

Materials

• U2OS human osteosarcoma cells (ATCC^®^HTB-96™)
• 35-mm tissue culture dishes
• Jet Pei (Polyplus Transfection, cat. no. 101-10N)
• pEGFP-γ-tubulin^334-449^ [6] (Addgene Plasmid # 87859)
• pTER-*γTUBULIN* shRNA [7] (Addgene Plasmid # 87955)
• 35-mm MatTek glass bottom dishes (MatTek corporation, cat. no. P35G-0.170-14-C)
• Zeocin (ThermoFisher Scientific, cat. no. R25001)
• Geneticin (G418; ThermoFisher Scientific, cat. no. 10131035)

Note that this list includes only necessary cell lines and non-standard laboratory equipment.

1. In a 35-mm tissue culture dish, U2OS cells are cultured and transfected at a ratio of 1:1 of pTER-*γTUBULIN* shRNA and pEGFP-γ-tubulin^334-449^ using Jet Pei according to the instructions of the cells distributors and the Jet Pei manufacturer.
2. Two days after transfection, stably transfected U2OS cells co-expressing *γTUBULIN* shRNA (reduces the expression of the endogenous γ-tubulin pool) and human GFP-tagged sh-resistant γ-tubulin C termini (GFP-γ-tubulin^334-449^), designated *γTUBULIN*sh-U2OS–GFP-γ-tubulin^334-449^ cells, are obtained by supplementing the cell medium with both 100 μg/mL zeocin and 200 μg/mL G418. Non-transfected cells will die within 2–3 days.
3. Add fresh medium supplemented with 100 μg/mL zeocin and 200 μg/mL G418 as deemed suitable. Passages to new cell culture dishes are not necessary until the 35-mm tissue culture dish is filled to capacity. It takes 2–3 weeks to obtain a stable cell line.
4. Once a stable *γTUBULIN*sh-U2OS–GFP-γ-tubulin^334-449^ cell line is established, plate 3 × 10^5^ *γTUBULIN*sh-U2OS–GFP-γ-tubulin^334-449^ cells on a 35-mm MatTek glass bottom dish 24 h before imaging.

*Step 2: cell imaging of live and fixed cells*

Materials

• Phosphate-buffered saline (PBS)
• Freshly made 4% paraformaldehyde and 2% sucrose dissolved in PBS (Psuc buffer); this solution can be stored at −20 °C for approximately 2–3 weeks
• 1:1 methanol, acetone solution (Met/Ac)
• A felt-tip pen
• A fluorescence/confocal microscope with a cell incubator equipped with a heating and CO_2_ system (necessary for incubations longer than 3 h)

Note that the following list includes only necessary non-standard laboratory equipment.

1. Before starting cell imaging, place Met/Ac at −80 °C in a freezer and prepare/thaw Psuc buffer.
2. Warm and CO_2_ equilibrate the microscope’s incubator 30 min before starting the imaging of the cells.
3. Place *γTUBULIN*sh-U2OS–GFP-γ-tubulin^334-449^ cells in a 35-mm MatTek glass bottom dish positioned in the dish holder of the microscope.
4. Find a suitable cell (Fig. 1A). Most cells in a stable *γTUBULIN*sh-U2OS–GFP-γ-tubulin^334-449^ cell line form tubular structures containing GFP-γ-tubulin^334-449^ (Fig. 1A) [2].

**Fig. 1:**
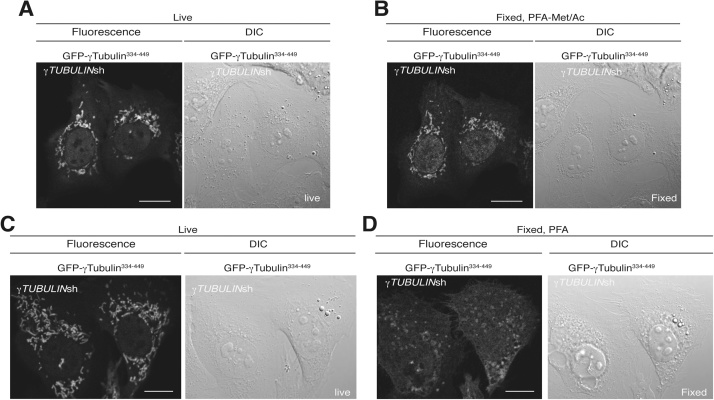
Examples of differential interference contrast (DIC) and confocal fluorescence images obtained simultaneously in a Zeiss LSM 700 Axio Observer microscope and showing the same two U2OS cells live (A) and after fixation 3 min with Psuc followed by Met/Ac (B) or live (C) and after fixation 5 min with Psuc (D). (A–D) These cells stably co-expressed *γTUBULIN* shRNA and GFP-γ-tubulin^334-449^, and in the images they exhibit GFP-γ-tubulin^334–449^-labelled structures. Scale bar: 10 μm.
5. Mark the location examined in the dish with a felt-tip pen so that the same position can be found again. An optional approach at this point is to remove the dish from the microscope and then put it back and re-examine it to find the same location again, and this procedure can be repeated until the same cell is found each time.
6. Drawing a mark on the plate may move the dish to some degree; find the chosen cell again and obtain a differential interference contrast (DIC) and a fluorescence (excitation max 488 nm, emission max 509 nm) image (Fig. 1A).
7. Rapidly remove the growth medium from the dish and replace it with 500 μL of Psuc buffer. Fix the cells for 3 min (Fig. 1A and B) at room temperature.
8. Remove the Psuc buffer and rapidly replaced Psuc buffer with 1 mL −80 °C Met/Ac. Immediately thereafter, incubate the dish for 3 min at −80 °C in a freezer.
9. Take the dish out of the freezer and remove the Met/Ac. Go to step 11.
10. If a final Met/Ac fixation and permeabilization step is not required, omit steps 7 to 9. Rapidly remove the growth medium from the dish and replace it with 500 μL of Psuc buffer. Fix the cells for 5 min (Fig. 1C and D) at room temperature.
11. Wash the cells twice with 1 mL of PBS.
12. Add 1 mL of PBS.
13. Put the dish containing the fixed cells in the microscope dish holder. Use the pen markings to place the plate in the approximately the same position as previously in the holder and find the previously imaged cell.
14. Search the sample thoroughly by starting at a certain point and moving up and down in the field of view. When lateral movements are necessary, make sure that the new microscope field partially overlaps with the previous one so that no area in the sample is overlooked. Keep searching until the same cell is found (Fig. 1A and C). Obtain a DIC and a fluorescence image (Fig. 1B and D). Compare the captured images of the fixed cell with the images of the live cell to ensure that the same cell has actually been identified.

Note that in comparison to live cells, the method that better preserves γ-tubulin-containing structures in fixed cells is the two-step fixation using paraformaldehyde followed by fixation with methanol/acetone (Fig. 1) [1,[3], [4], [5]].

Imaging of endogenous γ-tubules in fixed cells [1]

*Step 1: plating cells*

Materials

• U2OS human osteosarcoma cells (ATCC^®^HTB-96™)
• MCF10A human mammary gland epithelial cells (ATCC^®^CRL-10317™)
• 35-mm MatTek glass bottom dishes (MatTek corporation, cat. no. P35G-0.170-14-C) or 22 × 22 mm coverslips (MatTek corporation, cat. no. PCS-1.5-2222)
• 35-mm tissue culture dishes

Note that the following list includes only necessary cell lines and non-standard laboratory equipment.

1. U2OS and MCF10A cells are cultured according to the instructions of the cells distributors.
2. Plate 8 × 10^4^ U2OS or MCF10A cells in a 35-mm MatTek glass bottom dish 72 h before imaging. Alternatively, place a coverslip on the bottom of a 35-mm tissue culture dish and then plate 11 × 10^4^ U2OS or MCF10A cells in the dish. Ensure that the cell suspension is actually applied on the coverslip by gently pressing on the coverslip with the tip of the pipette during the plating of the cells in the dish; this step helps avoid growth of cells under the coverslip. The longer the plated cells grow in the dish, the more and the longer γ-tubules can be found [1].

*Step 2: cell imaging of live and fixed cells*

Materials

• PBS
• Freshly made 4% paraformaldehyde and 2% sucrose dissolved in PBS (Psuc buffer); Psuc buffer, which can be stored at −20 °C for an approximately 2–3 weeks
• 1:1 Met/Ac
• PBS staining buffer (PBSB) consisting of PBS, 1% fetal calf serum and 0.5% bovine serum albumin
• A fluorescence/confocal microscope
• One of the following anti-γ-tubulin antibodies: mouse or rabbit anti-γ-tubulin (1:400; Sigma-Aldrich, cat. no T6557, T3320 and T5192) or anti-γ-tubulin (1:400; Abcam, cat. no. ab27074)
• Optional primary antibodies: anti-α-tubulin (1:1000; Merck, cat. no. CP06) or anti-pericentrin (1:400; Atlas antibodies, cat. no. HPA016820)
• A secondary antibody: donkey anti-mouse (1:800; Jackson, cat. no. 715-545-150) or rabbit (1:800; Jackson, cat. no. 711-545-152) Alexa fluor 488 conjugated
• Optional secondary antibodies: donkey anti-mouse (1:1600; Jackson, cat. no. 715-005-150), rabbit (1:1600; Jackson, cat. no. 711-005-152) or Cyanine Cy3 conjugated
• Vectashield (Vector laboratories, cat. no. H-1000)
• Superfrost plus microslide (WWR, cat. no. 48311-703)
• Nail polish

1. Before starting the experiment, place Met/Ac at −80 °C in a freezer and prepare/thaw Psuc buffer.
2. Take the dish out of the incubator and rapidly remove the growth medium and replace it with 800 μL of Psuc buffer. Fix the cells for 3 min at room temperature.
3. Remove the Psuc buffer and rapidly replaced it with 1 mL of −80 °C Met/Ac. Immediately thereafter, incubate the dish for 3 min at −80 °C in a freezer.
4. Take the dish out of the freezer and remove the Met/Ac.
5. Wash the cells twice with 1 mL of PBS
6. To prevent non-specific antibody binding, incubate the sample in 600 μL of PBSB for 5 min.
7. Mix 1.25 μL of primary anti-γ-tubulin antibody with 500 μL of PBSB, and then incubate the cell sample with the primary antibody in buffer for 1 h.
8. Remove the primary antibody and wash the sample once with PBSB for 5 min.
9. Mix 0.6 μL of Alexa488-linked secondary antibody with 500 μL of PBSB and then incubate the sample with the secondary antibody in buffer for 1 h. Go to step 12.
10. If immunostaining of the cell sample with a second primary antibody is required, repeat step 7 and 8 with a second primary antibody. Used either 0.5 μL or 1.25 μL of primary anti-α-tubulin and anti-pericentrin antibody, respectively.
11. Mix 0.6 μL of Alexa488-linked and 0.3 μL Cy3-linked secondary antibodies with 500 μL of PBSB and then incubate the sample with the secondary antibodies in buffer for 1 h.
12. Remove the antibody/ies and wash the sample twice with PBSB for 5 min. If the cells were platted on a coverslip, go to step 15.
13. Remove the PBSB and add 1 mL of PBS to the 35-mm MatTek glass bottom dish.
14. Place the fixed cells in the microscope dish holder. Look for U2OS cells containing γ-tubules (Fig. 2A), centrosomes (Fig. 2B) and microtubules (Fig. 2C) [1].

**Fig. 2:**
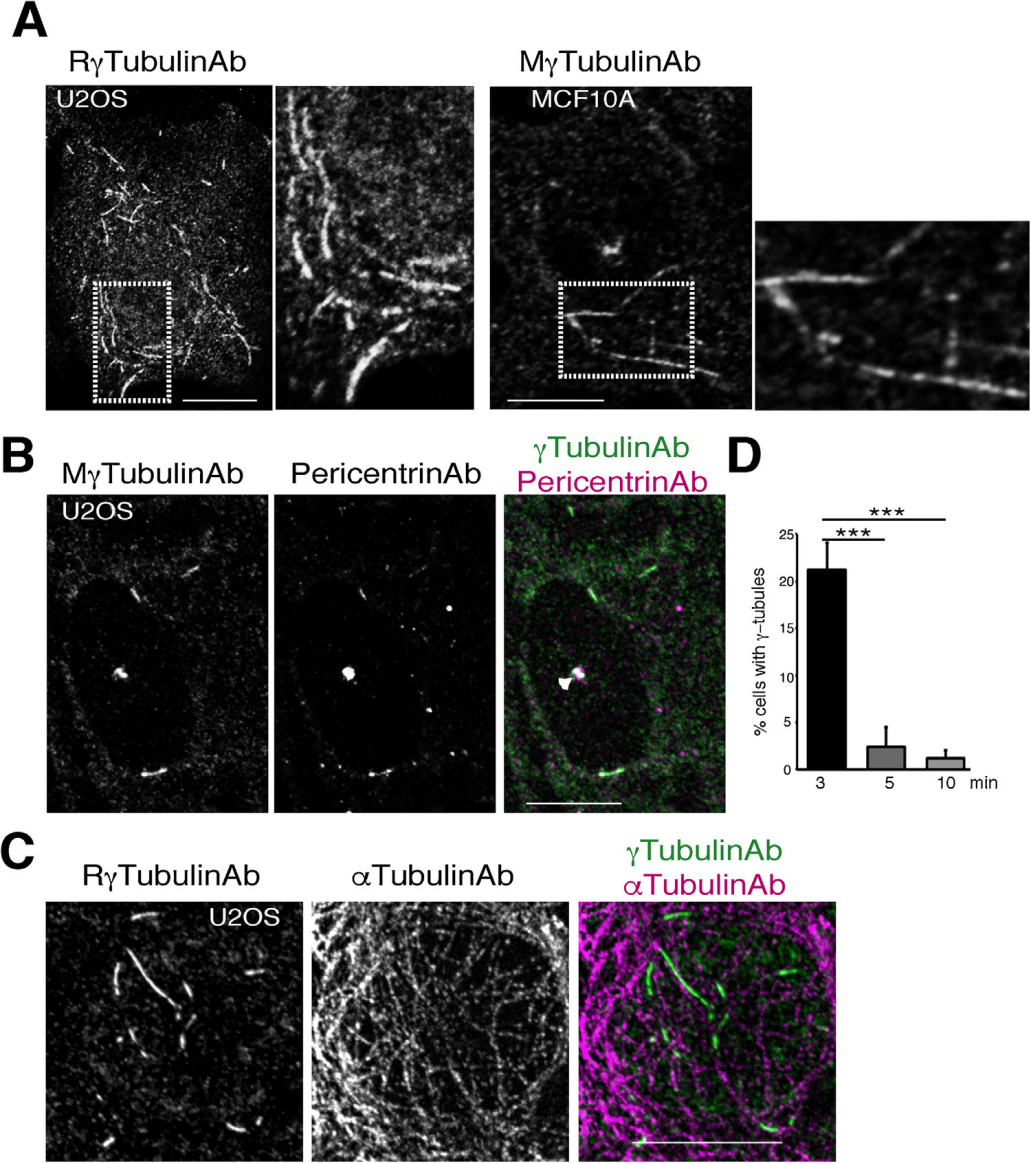
Confocal images of Psuc–Met/Ac-fixed cells. (A) A U2OS and a MCF10A cell with endogenous cytosolic γ-tubulin-containing structures (γ-tubules). The inset (white box) in the image to the left indicates the area shown in the image to the right, which illustrates γ-tubules at a higher magnification. (A–C) Cells were immunostained with an anti-γ-tubulin antibody produced in rabbit (RγTubulinAb, T3320) or mouse (MγTubulinAb, T6557) and co-stained with an anti-pericentrin (B) or an anti-α-tubulin antibody (C), as indicated. Scale bar: 10 μm. (B) The white arrowhead shows the centrosomes. (D) Graph showing the percentage of fixed cells with γ-tubules after fixation 3, 5 and 10 min with Psuc followed by Met/Ac fixation for 3, 5 and 10 min. A minimum of 100 cells was counted in each sample, and the percentage of cells with γ-tubules was calculated (mean ± SD; *N* *=* 5, *** *P* < 0.001).
15. If the cells were plated on a coverslip, place a drop of Vectashield on a slide and mount the coverslip. Wipe off excess of Vectashield with tissue paper and fix the coverslip to the slide with nail polish. Allow the nail polish to dry for 5 min.
16. Examine the slice to identify U2OS and MCF10A cells containing γ-tubules (Fig. 2).

## Additional information

Over the past decades, the functions of γ-tubulin have been studied, but very little is known about the dynamic and function of endogenous γ-tubules. With the above methodology, γ-tubules are preserved in fixed cells. Note that the longer duration of fixation, the fewer number of cells with γ-tubules (Fig. 2D).
